# Escalated Oxycodone Self-Administration and Punishment: Differential Expression of Opioid Receptors and Immediate Early Genes in the Rat Dorsal Striatum and Prefrontal Cortex

**DOI:** 10.3389/fnins.2019.01392

**Published:** 2020-01-09

**Authors:** Christopher A. Blackwood, Michael T. McCoy, Bruce Ladenheim, Jean Lud Cadet

**Affiliations:** Molecular Neuropsychiatry Research Branch, NIH/NIDA Intramural Research Program, Baltimore, MD, United States

**Keywords:** oxycodone, opioid receptors, protein, mRNA, footshocks, prefrontal cortex, dorsal striatum

## Abstract

Opioid use disorder (OUD) is characterized by compulsive drug taking despite adverse life consequences. Here, we sought to identify neurobiological consequences associated with the behavioral effects of contingent footshocks administered after escalation of oxycodone self-administration. To reach these goals, Sprague-Dawley rats were trained to self-administer oxycodone for 4 weeks and were then exposed to contingent electric footshocks. This paradigm helped to dichotomize rats into two distinct behavioral phenotypes: (1) those that reduce lever pressing (shock-sensitive) and (2) others that continue lever pressing (shock-resistant) for oxycodone during contingent punishment. The rats were euthanized at 2-h after the last oxycodone plus footshock session. The dorsal striata and prefrontal cortices were dissected for use in western blot and RT-qPCR analyses. All oxycodone self-administration rats showed significant decreased expression of Mu and Kappa opioid receptor proteins only in the dorsal striatum. However, expression of Delta opioid receptor protein was decreased in both brain regions. RT-qPCR analyses documented significant decreases in the expression of *c-fos*, *fosB*, *fra2*, *junB*, *egr1*, and *egr2* mRNAs in the dorsal striatum (but not in PFC) of the shock-sensitive rats. In the PFC, *junD* expression was reduced in both phenotypes. However, *egr3* mRNA expression was increased in the PFC of only shock-resistant rats. These results reveal that, similar to psychostimulants and alcohol, footshocks can dichotomize rats that escalated their intake of oxycodone into two distinct behavioral phenotypes. These animals also show significant differences in the mRNA expression of immediate early genes, mainly, in the dorsal striatum. The increases in PFC *egr3* expression in the shock-resistant rats suggest that Egr3 might be involved in the persistence of oxycodone-associated memory under aversive conditions. This punishment-driven model may help to identify neurobiological substrates of persistent oxycodone taking and abstinence in the presence of adverse consequences.

## Introduction

Opioid use disorders (OUDs) continue to constitute a public health crisis (Cicero et al., 2005; Skolnick, 2017). OUDs are characterized by compulsive consumption of large amounts of opioids despite adverse life consequences (American Psychiatric Association and Dsm-5 Task Force, 2013). Adverse consequences of OUDs are influenced by widespread use of opioids such as oxycodone for the treatment of various pain syndromes (Gaskell et al., 2016; Schmidt-Hansen et al., 2017) and their over-prescription by medical professionals (Van Zee, 2009). These have led to substantial increases in the number of deaths caused by opioid overdoses (Skolnick, 2017). To develop more effective treatment strategies for OUDs (Blanco and Volkow, 2019; Patel and Kosten, 2019), animal models that meet multiple DSM criteria are needed to increase their translational validity (Cadet, 2019).

Efforts to elucidate the molecular neurobiology of substance use disorders (SUDs), including OUDs, have largely depended on drug self-administration (SA) models (Wade et al., 2015; Mavrikaki et al., 2017; Blackwood et al., 2018). However, as suggested by several investigators (Chen et al., 2013; Belin-Rauscent et al., 2016; Cadet, 2019), drug self-administration alone represents only one DSM criterion and fails to replicate the complex behavioral syndromes termed SUDs (American Psychiatric Association and Dsm-5 Task Force, 2013). Some groups have tried to remedy this conundrum by adding contingent punishment during the performance of self-administration of alcohol, cocaine, and methamphetamine by rodents (Chen et al., 2013; Vanderschuren et al., 2017; Marchant et al., 2018; Cadet et al., 2019). In these models, footshocks are used to segregate rats that continue to self-administer drugs [shock-resistant (SR), addicted] from those that significantly reduce or stop [shock-sensitive (SS), non-addicted users] their intake in the presence of punishment (Chen et al., 2013; Vanderschuren et al., 2017; Marchant et al., 2018; Cadet et al., 2019). Those rats that significantly reduced or stopped their drug intake may represent a group of individuals who use illicit drugs without meeting DSM criteria for SUD (Korf et al., 2010; Zaaijer et al., 2014). Similar to observations with psychostimulants, rats, given long access to oxycodone, escalate their intake and exhibit compulsive drug seeking behaviors (Wade et al., 2015; Blackwood et al., 2018; Bossert et al., 2018). However, it remains to be determined if punishment could also help to dichotomize rats that escalated their oxycodone intake into SR and SS rats as reported for other drugs (Chen et al., 2013; Marchant et al., 2018; Cadet et al., 2019).

Behaviors measured during drug self-administration have been shown to depend on interactions of interconnected brain regions that include the dorsal striatum and the prefrontal cortex (PFC) among others (Everitt, 2014; Moorman et al., 2015; Smith and Laiks, 2018). On the one hand, the dorsal striatum has been reported to play prominent roles in the mediation of habitual drug taking behaviors (Belin and Everitt, 2008; Everitt and Robbins, 2016; Hodebourg et al., 2018). On the other hand, the PFC is involved in drug seeking, reinstatement of drug seeking, and other complex cognitive behaviors including decision making in relation to drug taking behaviors (Bossert et al., 2011; Perry et al., 2011; Hu et al., 2019). Human studies have also documented abnormalities in these regions of humans who suffer from SUDs (Bolla et al., 2004; Goldstein and Volkow, 2011; Cadet et al., 2014; Cadet and Bisagno, 2015). Of further relevance to investigations of oxycodone use disorder and other OUDs, in general, is the fact that these brain regions contain high concentration of opioid receptors. For example, using receptor autoradiographic techniques, Mansour et al. (1987) had reported large concentration of mu and delta opioid receptors in the frontal cortex and dorsal striatum of rats. The dorsal striatum also contains high concentration of kappa opioid receptors (Mansour et al., 1987). These regions also contain mRNAs that code for these receptors (Mansour et al., 1994). Human studies using PET and radiolabeled carfentanil have also identified mu opioid receptors in these brain regions (Frost et al., 1985; Nummenmaa et al., 2018; Burns et al., 2019). Of significance to the presence study, post-mortem studies have reported decreased expression of mu opioid receptors in the striatum (Sillivan et al., 2013) and PFC of heroin abusers (Ferrer-Alcon et al., 2004b). Other post-mortem abnormalities in signaling pathways have also been reported in the PFC of opioid users (Ferrer-Alcon et al., 2004a; Keller et al., 2017).

Thus, we reasoned that biochemical and molecular adaptations in these brain regions might play important roles in the behavioral manifestations of punishment-induced separation of rats into resistant and sensitive rats. This idea is supported by our recent demonstration that decreases in the expression of Mu opioid receptor protein levels in the dorsal striatum correlated with escalated drug intake and drug seeking behavior in oxycodone self-administering rats (Blackwood et al., 2018). This reasoning is also consonant with our findings of dose-dependent changes in the mRNA expression of immediate early genes (IEGs) following oxycodone seeking after a month of withdrawal from escalated oxycodone SA (Blackwood et al., 2019). We reasoned further that shock-resistant and shock-sensitive rats may show differential expression of opioid receptors in the dorsal striatum and of IEGs in both the dorsal striatum and PFC. Changes in IEG expression have been reported in several models of psychostimulant and OUDs (Bisagno and Cadet, 2019), supporting the possibility that IEGs could be used as markers of the two shock-induced divergent phenotypes.

As predicted, footshocks did help to separate oxycodone SA rats into SR and SS phenotypes. These two phenotypes showed differential expression of IEGs in the dorsal striatum and PFC, with greater changes occurring in the striatum. These data further implicate the dorsal striatum as a major node in the chemical neuroanatomical pathways of habitual compulsive oxycodone taking.

## Materials and Methods

### Subjects

Male Sprague-Dawley rats (Charles River, Raleigh, NC, United States), weighing 350–400 g before surgery, were used in all experiments. Rats were maintained on a 12-h reversed light/dark cycle with food and water available *ad libitum*. This study was carried out in accordance with the principles and guidelines outlined in the National Institutes of Health (NIH) Guide for the Care and Use of Laboratory Animals (eighth Edition^1^) as approved by the NIDA (National Institute of Drug Abuse) Animal Care and Use Committee at the Intramural Research Program (IRP).

### Intravenous Surgery and Self-Administration Training

Surgical implantations of intravenous catheters were done as previously described (Cadet et al., 2017). Briefly, we anesthetized the rats with an intraperitoneal injection of ketamine (50 mg/kg) and xylazine (5 mg/kg) and inserted a polyurethane catheter (SAI Infusion Technologies, Lake Villa, IL, United States) into the jugular vein. One end of the catheters was inserted in the jugular vein while the other end was attached to a modified 22-gauge cannula (Plastics One, Roanoke, VA, United States) that was mounted to the back of each rat. The modified cannulae were connected to a fluid swivel (Instech, Plymouth, PA, United States) via polyethylene-50 tubing that was protected by a metal spring. These cannulae served as infusion ports for the catheters. The ports were closed using dust caps (PlasticOne, Roanoke, VA, United States). Thereafter, the catheters were flushed every 48-h with gentamicin (0.05 mg/kg, Henry Schein, Melville, NY, United States) in sterile saline to maintain patency. Injections of buprenorphine (0.1 mg/kg) were used post-surgery to relieve pain.

Rats were allowed to recover for 5–7 days before oxycodone self-administration (SA) training. Rats were trained in SA chambers located inside sound-attenuated cabinets and controlled by a Med Associates System (Med Associates, St Albans, VT, United States). Each chamber was equipped with two levers located 8.5 cm above the grid floor. Presses on the retractable active lever activated the infusion pump and tone-light cue. Presses on the inactive lever had no reinforced consequences.

### Training and Punishment Phases

**FIGURE 1:**
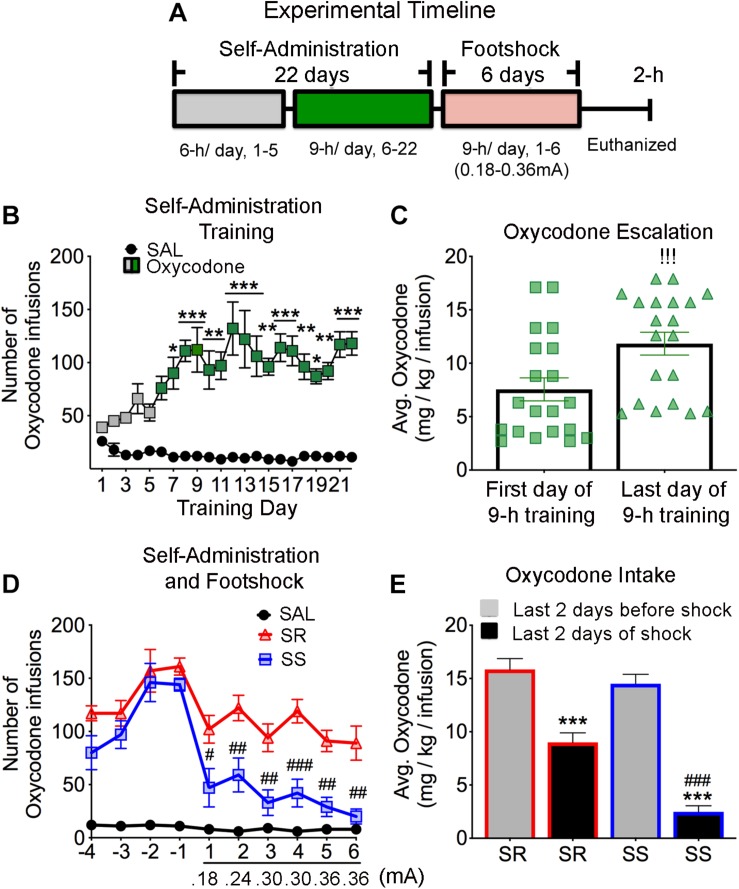
Long access to oxycodone self-administration and contingent punishment dichotomize rats into shock-resistant and shock-sensitive phenotypes. **(A)** Experimental timeline of oxycodone self-administration training and footshock phases. Rats were trained to self-administer oxycodone using a long access (LgA) paradigms of 6-h for 1–5 days, 9-h for 6–22 days, followed by oxycodone SA and contingent footshocks (0.18–0.36 mA) for 9-h for 6 more days. **(B)** All rats escalate their intake of oxycodone during long access self-administration training (*n* = 20). **(C)** During the 9-h training paradigm rats showed significantly higher levels of oxycodone on the last day compared to the first day (*n* = 20). **(D)** Footshocks caused decreased lever pressing significantly more in the shock-sensitive (SS) than in the shock-resistant (SR) rats (*n* = 5, SS; *n* = 6, SR). **(E)** SS rats took substantially less oxycodone than SR rats. Key to statistics: ^∗^, ^∗∗^, ^∗∗∗^*p* < 0.05, 0.01, 0.001, respectively, in comparison to saline rats or last 2 days before shocks in the SR subgroup; #, ##, ###*p* < 0.05, 0.01, 0.001, respectively, in comparison to SR rats or last 2 days of shocks in the SR subgroup. !!!*p* < 0.001, in comparison to first day of 9-h training. Stats were performed by two-way ANOVA followed by Bonferroni *post hoc* tests or Student’s *t*-test.

Rats (*n* = 28) were housed in Med Associates SA chambers and were randomly assigned to either saline (Sal) (*n* = 8) or oxycodone (*n* = 20) conditions. Rats were given long access to oxycodone and were trained for two 3-h sessions during days 1–5, followed by three 3-h sessions during days 6–22 (Figure 1A). Each of the 3-h sessions was separated by 30-min intervals during which rats remained in operant chambers but had no access to the levers to press for oxycodone. Lever presses were reinforced using a fixed ratio-1 with a 20-s timeout accompanied by a 5-s compound tone-light cue. Rats self-administered oxycodone at a dose of 0.1 mg/kg per infusion given over 3.5-s (0.1 ml per infusion). The house light was turned off and the active lever retracted at the end of the 3-h session. After training rats for 22 days, oxycodone rats that escalated their oxycodone intake equal to or greater than 50 daily infusions underwent the punishment phase.

During the punishment phase, rats continued to self-administer oxycodone every day (three 3-h sessions/day separated by 30 min off intervals) as described above. During that phase, ∼50% of the reinforced lever-presses also resulted in the delivery of a 0.5-s footshock through the grid floor. We set the initial footshock at 0.18 mA and increased the shock intensity by 0.06 mA to a final value of 0.36 mA over the course of 6 punishment days.

### Collection of Tissues

We euthanized rats 2-h after the last oxycodone plus punishment day. Brains were removed from skulls and the dorsal striata and PFC were dissected and snap frozen on dry ice. Dissections were performed as previously described (Blackwood et al., 2018). In brief, we used stereotaxic coordinates to dissect the dorsal striatum (A/P + 2 to −2 mm bregma, M/L ± 2 to 5 mm, D/V −3 to −6 mm) and the PFC (A/P + 2.7 to + 1.7 mm bregma, M/L 0 to + 4 mm, D/V + 7 to + 9 mm) using the Atlas by Paxinos and Watson (1998). We also followed distinguishable anatomical structures (olfactory bulb, corpus callosum, and lateral ventricle) for further accuracy. Tissues were then processed for western blotting and quantitative RT-PCR analyses.

### Quantitative RT-PCR

Total RNA was isolated from one hemisphere of the dorsal striatum and PFC using RNeasy Mini Kit (Qiagen, Valencia, CA, United States). Total RNA (0.5 μg) was reverse-transcribed (RT) with oligo dT primers using Advantage RT-for-PCR kit (Clontech, Mountain View, CA, United States). RT-qPCR was performed as previously described (Cadet et al., 2017; Blackwood et al., 2019) with Roche LightCycler 480 II (Roche Diagnostics, Indianapolis, IN, United States) using iQ SYBR Green Supermix (Bio-Rad, Hercules, CA, United States). Custom-designed primers were made and HPLC-purified by the Synthesis and Sequence Facility of the Johns Hopkins University (Baltimore, MD, United States). Primer sequences are listed in Table 1.

**TABLE 1:** List of RT-qPCR primers.

**Gene name**	**Forward**	**Reverse**
*c-fos*	GGG CAA AGT AGA GCA G	CTC TTT CAG TAG ATT GGC A
*fosB*	GAA GCT GGA GTT CAT GC	ATG GGC TTG ATG ACA GA
*fra1*	CAC CCT CTC TGA CTC CTT TTA	GAT TAA CAG GGA AAG GAG ATG A
*fra2*	TGC AAA ATC AGT CCT GAG GAA	CAA TGC TAA TGG GCT TGA TGA
*c-jun*	TCT CAG GAG CGG ATC AA	TGT TAA CGT GGT TCA TGA C
*junB*	TCT TTC TCT TCA CGA CTA CA	CTA GCT TCA GAG ATG CG
*junD*	GGA TTG AAA CCA GGG TC	TAG AGG AAC TGC GTA CT
*egr1*	TGC ACC CAC CTT TCC TAC TC	AGG TCT CCC TGT TGT TGT GG
*egr2*	CCT GAG ACC TCG AAA GTA	CAG ATC CGA CAC TGG AA
*egr2* (PFC)	CAA GGC CGT AGA CAA AAT CCC A	CCC ATG TAA GTG AAG GTC TGG T
*egr3*	AAA GAA GGG ATC TGA GAG GCG GAT	TGT GAG TTC TGT GGG CGC AAG TTT
*egr3* (PFC)	TGT AAT GGA CAT CGG TCT G	GGC TAA TGA TGT TGT CCT G
*b2m*	GAT CTT TCT GGT GCT TGT	AGC TCA ATT TCT ATT TGA GGT

### Western Blotting

Western blotting was performed essentially as previously described (Blackwood et al., 2018). In brief, soluble protein lysates were prepared in solutions that contained 1x NuPage LDS Sample Buffer (Thermo Fisher Scientific, Waltham, MA, United States), and 1% β-Mercaptoethanol (Sigma, St. Louis, MO, United States). Samples were then boiled and resolved using NuPage 10% Bis-Tris Protein Gels (Thermo Fisher Scientific, Waltham, MA, United States). Proteins were electrophoretically transferred onto Trans-Blot^®^ Turbo^TM^ Midi Nitrocellulose membranes using the Trans-Blot^®^ Turbo^TM^ system (Bio-Rad, Hercules, CA, United States). Primary rabbit antibodies used were anti-OPRM1 (1:5000, ab17934), anti-OPRK1 (1:10000, ab183825), anti-OPRD1 (1:1000, ab176324), and anti-Cyclophilin B (CYPB) (1:10000, ab16045) purchased from Abcam (Cambridge, MA, United States). Secondary antibodies used were goat anti-rabbit (1:500, Sc-1404) conjugated HRP purchased from Santa Cruz Biotechnology (Dallas, TX, United States). Following secondary antibody incubation, ECL clarity (Bio-Rad, Hercules, CA, United States) was used to detect gel bands on ChemiDoc Touch Imaging System (Bio-Rad, Hercules, CA, United States), and intensities were quantified with Image Lab version 6.0 (Bio-Rad, Hercules, CA, United States) software.

### Statistical Analyses

Behavioral data were analyzed using two-way analysis of variance (ANOVA). Dependent variables were the number of oxycodone infusions on training days. Independent variables were between-subject factor reward types (saline, Oxy, SR, SS), within-subject factor SA day (training days 1–22 or shock days 1–6), and their interactions. If the main effects were significant (*p* ≤ 0.05), Bonferroni *post hoc* tests were used to compare reward types on each training/shock day. Biochemical data were analyzed using one-way ANOVA followed by the Fisher’s PLSD *post hoc* test if the main effect was significant. Genes that showed a trend toward significance using ANOVA were also analyzed by Student’s *t*-test. Linear regression analyses were performed to detect potential relationships between the total amount of oxycodone taken during the last 3 days of footshocks and protein/mRNA expression. The null hypothesis was rejected at *p* ≤ 0.05. Behavioral data were analyzed with SPSS version 24 (IBM, Armonk, NY, United States), Prism 8.2.0 (GraphPad Software, San Diego, CA, United States) while biochemical data were analyzed using StatView version 4.0 (SAS, Cary, NC, United States).

## Results

### Footshocks Separate Oxycodone Self-Administering Rats Into Resistant and Sensitive Phenotypes

Figure 1 shows the timeline and results of the behavioral studies. The repeated-measures ANOVA for reward earned included the between-subject factor group (Saline and oxycodone) and the within-subject factor of SA day (training days 1–22), and the group × day interaction. This analysis showed statistically significant effects of group [*F*(1,572) = 272.8, *p* < 0.0001], non-significant effects of day [*F*(21,572) = 1.133, *p* = 0.3081], and significant group × day interaction [*F*(21,572) = 1.756, *p* = 0.0201]. Oxycodone rats increased their drug intake substantially after training day 6 when compared to saline rats [*F*(1,416) = 230.6, *p* = 0.0001] (Figure 1B). There was also a significant uptake of oxycodone between day 6 and day 22 during the 9-h training period (Figure 1C).

Similar to our previous reports (Cadet et al., 2016; Krasnova et al., 2017; Torres et al., 2017), the introduction of footshocks allowed for the separation of rats that had escalated their oxycodone intake into (1) shock-resistant (SR) animals that continue to press the lever slightly less than before and (2) shock-sensitive (SS) that markedly decreased their intake of the drug (Figure 1D). Fifty five percent (55%) of the oxycodone SA rats were classified as SR while 45% were denoted SS animals. Respectively, SR and SS exhibited 38 and 78% suppression of drug infusion during the last 2 days of footshocks in comparison to the last 2 days before the introduction of shocks (Figure 1D), indicating different degrees of suppression of individual oxycodone intake by the contingent shocks. For these data, the repeated measures ANOVA for reward earned included the SR and SS rats, and the within-subject factor days of footshocks (1–6 days), and the group × day interaction. We found statistically significant effects of shocks [*F*(1,54) = 75.0, *p* < 0.0001] and day [*F*(5,54) = 2.4, *p* = 0.0484], and non-significant effect of group × day interaction [*F*(5,54) = 0.1, *p* = 0.9857] (Figure 1D). A similar ANOVA model for reward earned prior to the punishment phase found statistically significant effects of group [*F*(1,196) = 40.25, *p* < 0.0001], day [*F*(21,196) = 4.369, *p* < 0.0001], and non-significant group × day interaction [*F*(21,196) = 0.9484, *p* = 0.2076]. Further analyses revealed no significant differences between SS and SR rats during training days 1–22. We also compared the amount of oxycodone taken during the last 2 days of SA training alone to the amount taken during the phase of oxycodone SA plus footshocks with shock intensity at 0.36 mA. Figure 1E shows that the amount of oxycodone (mg/kg) consumed during the last 2 days of punishment was significantly decreased in both SR and SS rats [*F*(3,36) = 41.30, *p* < 0.0001], with the SR rats consuming much more oxycodone than the SS rats.

### Effects of Oxycodone Self-Administration and Footshocks on Opioid Receptor Protein Expression

Oxycodone exerts its actions by binding to opioid receptors in the brain (Williams et al., 2013). We had previously reported that rats that escalated their intake of oxycodone showed decreased Mu opioid receptor protein levels in the dorsal striatum even after a month of withdrawal (Blackwood et al., 2018). In contrast, oxycodone-exposed rats showed comparable expression of Delta and Kappa opioid receptor proteins to control rats (Blackwood et al., 2018). Here, we tested the possibility that there might be differences in the expression of the 3 opioid receptors in the dorsal striatum and PFC of rats euthanized at 2-h after the last session.

**FIGURE 2:**
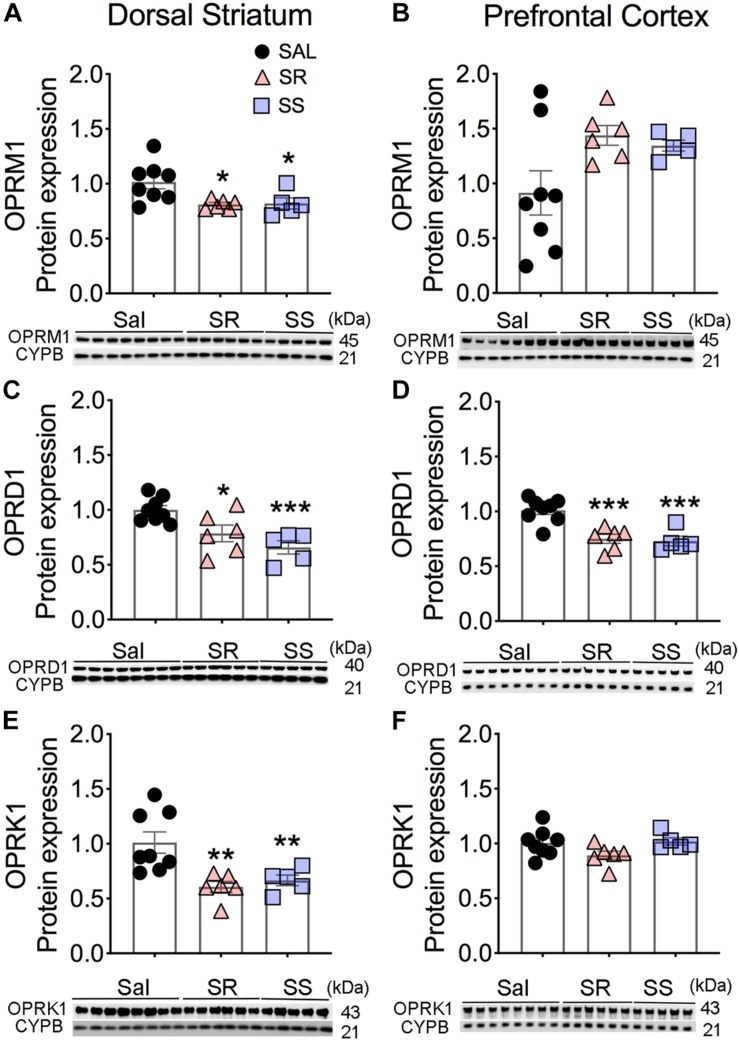
Differential expression of Mu, Delta, and Kappa opioid receptor proteins in the dorsal striatum and prefrontal cortex (PFC) of oxycodone- and shock-exposed rats. **(A–F)** Quantitative measures and representative images showing Western blot analyses of Mu (OPRM1), Delta (OPRD1), and Kappa (OPRK1) in the SR and SS rats show decreased OPRM1 **(A)**, OPRD1 **(C)**, and OPRK1 **(E)** in the dorsal striatum. There were also significant decreases in OPRD1 **(D)**, but not of OPRM1 **(B)** and OPRK1 **(F)**, receptor protein levels in the PFC (*n* = 8, Sal; *n* = 6, SR; *n* = 5, SS). Key to statistics: ^∗^, ^∗∗^, ^∗∗∗^*p* < 0.05, 0.01, 0.001, respectively, in comparison to saline-exposed rats or SR rats. Stats were performed by one-way ANOVA and Fisher’s PLSD *post hoc* tests.

There were significant decreases in striatal Mu (OPRM1) [*F*(2,16) = 5.352, *p* = 0.0166], Delta (OPRD1) [*F*(2,16) = 15.45, *p* = 0.0002], and Kappa (OPRK1) [*F*(2,16) = 8.230, *p* = 0.0035] protein levels in both SR and SS rats compared to the saline SA animals (Figures 2A,C,E). In contrast, there were no substantial changes in the protein expression of OPRM1 (*p* = 0.0837) and OPRK1 (*p* = 0.1030) in the PFC (Figures 2B,F). However, significantly decreases in OPRD1 [*F*(2,16) = 9.219, *p* = 0.0022] protein levels were observed in both SR and SS subgroups compared to the saline group (Figure 2D) in the PFC. These data suggest region-specific regulation of opioid receptor expression in the brain.

### Effects of Oxycodone SA and Footshocks on Members of Fos Family of IEGs

#### Dorsal Striatum

**FIGURE 3:**
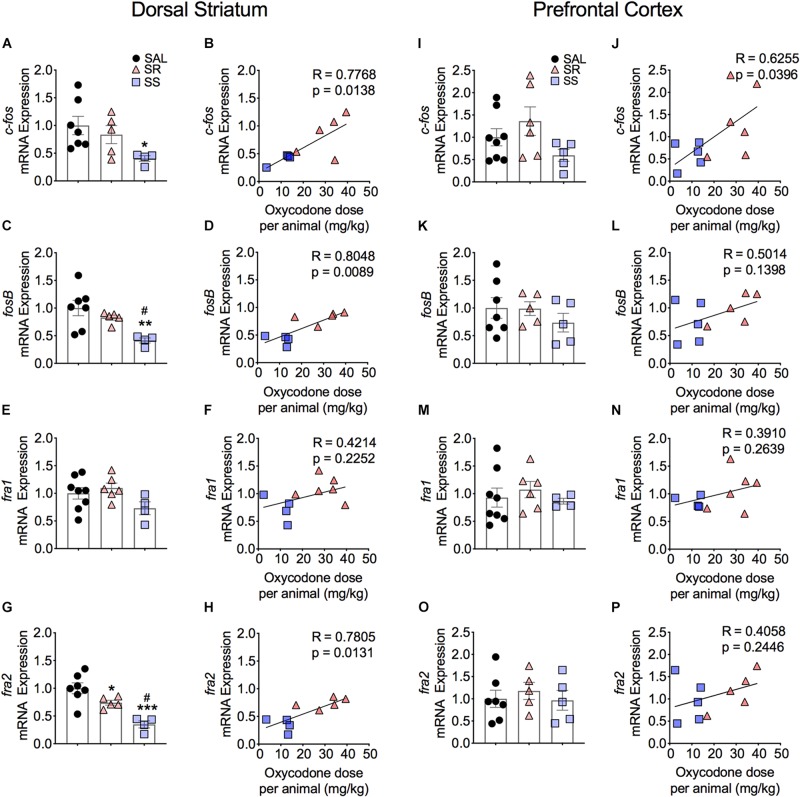
Striatal Fos Family members show decreased mRNA expression. SS rats show decreased striatal *c-fos* **(A)**, *fosB* **(C)**, and *fra2* **(G)** mRNA levels. mRNA expression of *c-fos*, *fosB*, and *fra2* correlated with doses of oxycodone **(B,D,H)**. Striatal *fra1* **(E,F)** show no significant changes in mRNA expression. No significant changes in *c-fos* **(I)**, *fosB* **(K,L)**, *fra1* **(M,N)**, and *fra2* **(O,P)** mRNA levels were detected in the PFC. The expression of *c-fos* correlated with doses of oxycodone **(J)** (*n* = 7–8, Sal; *n* = 5–6, SR; *n* = 4–5, SS). Key to statistics: ^∗^, ^∗∗^, ^∗∗∗^*p* < 0.05, 0.01, 0.001, respectively, in comparison to saline rats. #*p* < 0.05, in comparison to SR rats. Stats were performed by one-way ANOVA, Fisher’s PLSD *post hoc*, or Student’s *t*-test.

As noted in the introduction, because the expression of several IEGs is known to be impacted by administration of opioid drugs (Bisagno and Cadet, 2019; Blackwood et al., 2019), we reasoned that differential expression of some IEGs of the Fos family might occur in the SR and SS oxycodone SA phenotypes, with the SR showing higher mRNA expression. Figure 3 shows the effects of footshocks and oxycodone SA on *c-fos*, *fosB*, *fra1*, and *fra2* mRNA levels in the dorsal striatum of the SR and SS rats. We found that the expression of *c-fos* mRNA levels showed a trend toward significance [*F*(2,13) = 3.47, *p* = 0.0621]. Planned *t*-test between the oxycodone-exposed rats and control rats revealed significant differences in *c-fos* mRNA expression between the SS subgroup compared to controls (*p* = 0.0212) but not compared to the SR subgroup (Figure 3A). Regression analysis revealed that the levels of mRNA expression were significantly related to the amount of total oxycodone taken during the experiment (Figure 3B). We also observed significant decreases in striatal mRNA levels of *fosB* [*F*(2,13) = 6.554, *p* = 0.0108] (Figure 3C) and *fra2* [*F*(2,13) = 14.326, *p* = 0.0005] (Figure 3G), but not of *fra1* (Figure 3E) in SS rats in comparison to control and SR rats. The *fra2* mRNA levels also showed significant decreases in the SR rats compared to saline rats. The mRNA expression levels of *c-fos*, *fosB* and *fra2* also showed significant linear correlation with total oxycodone taken (Figures 3B,D,H) whereas *fra1* expression did not show any significant correlation with oxycodone SA (Figure 3F).

#### Prefrontal Cortex

Figure 3 also illustrates the results of footshocks on *c-fos*, *fosB*, *fra1*, and *fra2* mRNA levels in the PFC. In the PFC, although the mRNA expression of *c-fos* did trend toward significance (*p* = 0.0701) between the SR and SS subgroups, we detected no significant changes (Figure 3I). However, the regression analysis revealed significant relationship of *c-fos* mRNA expression to amount of oxycodone taken (Figure 3J). There was no significant change in *fosB*, *fra1* or *fra2* (Figures 3K,M,O). Moreover, regression analysis revealed no significant correlations between these genes expression and the amount of oxycodone taken (Figures 3L,N,P).

### Differential Expression of Members of Jun IEG Family in the Dorsal Striatum and PFC of Oxycodone Exposed Rats

#### Dorsal Striatum

**FIGURE 4:**
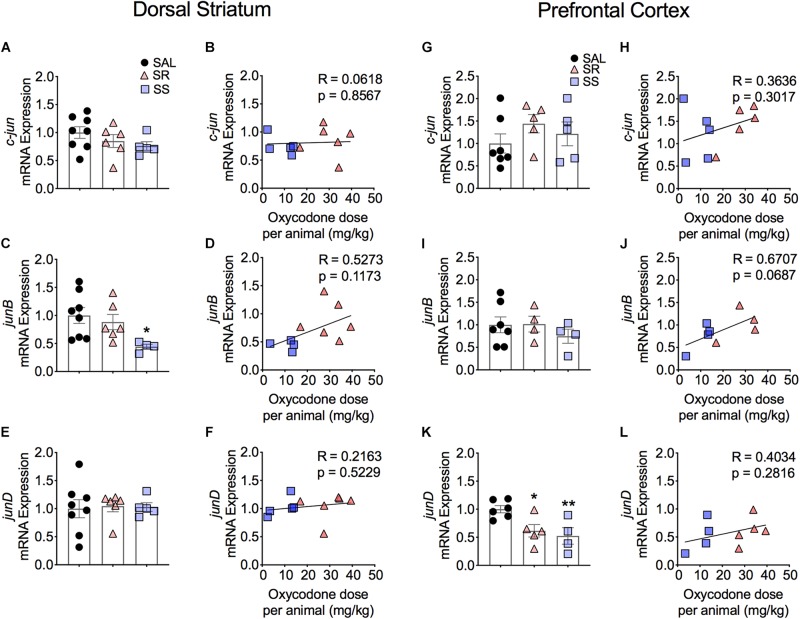
Striatal *junB and* cortical *junD* mRNA expression is significantly decreased in shock-sensitive rats. There were no significant changes in mRNA levels of *c-jun* in the dorsal striatum **(A,B)** and PFC **(G,H)**. SS rats show decreased *junB* in the dorsal striatum **(C)** but not in the PFC **(I)**. No significant correlations between *junB* and doses of oxycodone **(D,J)**. The expression of *junD* mRNA was not affected in the dorsal striatum **(E)** but was significantly decreased in the PFC of both SR and SS rats **(K).** There was no significant correlation in *JunD* **(F,L)** (*n* = 7–8, Sal; *n* = 5–6, SR; *n* = 4–5, SS). Key to statistics: ^∗^, ^∗∗^*p* < 0.05, 0.01, respectively, in comparison to saline rats. Stats were performed by one-way ANOVA and Fisher’s PLSD *post hoc* tests.

Figure 4 shows the observations for JUN family of IEGs in the dorsal striatum. The mRNA levels of *c-jun* or *junD* showed no significant changes between the subgroups (Figures 4A,E) and no correlation to oxycodone doses (Figures 4B,F). In contrast, we observed significantly decreased mRNA levels of *junB* [*F*(2,15) = 3.743, *p* = 0.0480) in SS rats compared to controls (Figure 4C). However, there was no correlation between mRNA expression of *junB* and the amount of oxycodone taken (Figure 4D).

#### Prefrontal Cortex

Figure 4 also shows the quantification of JUN family IEGs in the PFC. The mRNA expression of *c-jun* and *junB* showed no significant changes (Figures 4G,I) and no correlation to the total amount of infused oxycodone (Figures 4H,J). In contrast, we found significantly decreased mRNA levels of *junD* in the SR and SS rats [*F*(2,12) = 6.331, *p* = 0.0133] (Figure 4K). However, there was no relationship between the mRNA expression of *junD* and infused oxycodone (Figure 4L).

### Effects of Oxycodone SA and Footshocks on the Expression of Members of Egr Family of IEGs

#### Dorsal Striatum

**FIGURE 5:**
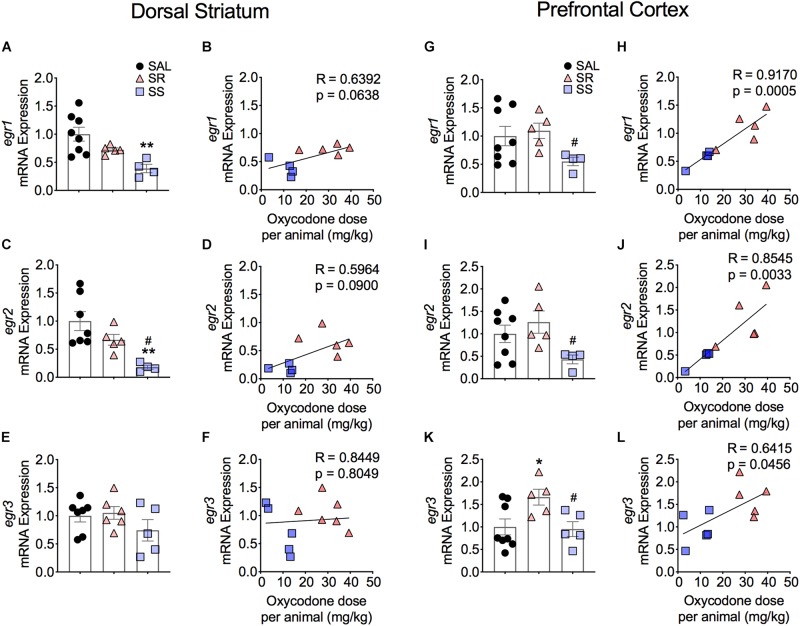
Differential expression of Egr family of IEGs in the dorsal striatum and PFC of shock-resistant and shock-sensitive rats. SS rats show decreased striatal *egr1* **(A)** and *egr2* **(C)** mRNA levels. There were no significant changes in mRNA levels of *egr1* **(B)** and *egr2* **(D)** in the PFC. Striatal *egr3* **(E)** showed no changes in mRNA expression in either SR or SS rats. SR rats show increased *egr3* **(F)** mRNA levels of in the PFC. SS rats showed significant correlation and decreased mRNA levels of *egr1* **(G,H)**, *egr2* **(I,J)**, and *egr3* **(K,L)** in the PFC (*n* = 7–8, Sal; *n* = 5–6, SR; *n* = 4–5, SS). Key to statistics: ^∗^, ^∗∗^*p* < 0.05, 0.01, respectively, in comparison to saline. #*p* < 0.05, in comparison to SR rats. Stats were performed by one-way ANOVA and Fisher’s PLSD *post hoc* tests.

Figure 5 shows the effects of footshocks from oxycodone SA on *egr1*, *egr2*, and *egr3* mRNA levels in the dorsal striatum. There were significantly decreases in mRNA levels of striatal *egr1* [*F*(2,14) = 7.588, *p* = 0.0059] and *egr2* [*F*(2,13) = 8.019, *p* = 0.0054] in the SS rats compared to saline rats (Figures 5A,C). Striatal *egr2* expression in the SS rats was also significantly decreased in comparison to SR rats (Figure 5C). We found no changes in *egr3* mRNA expression (Figure 5E). Moreover, there were no significant correlations between mRNA levels of *egr1*, *egr2, and egr3* and the amount of oxycodone taken (Figures 5B,D,F).

#### Prefrontal Cortex

Figure 5 also illustrates the results of Egr family of IEGs in the PFC. There were significantly decreases in mRNA levels of *egr1* (*p* = 0.0141) and *egr2* (*p* = 0.0256) in the SS rats compared to SR rats (Figures 5G,I). These changes showed a significant correlation between the mRNA expression and the amount of oxycodone taken (Figures 5H,J). In addition, we detected significant increases in *egr3* [*F*(2,13) = 4.314, *p* = 0.0331] in the SR rats in comparison to both saline rats and SS rats (Figure 5K). A significant correlation was also found between the mRNA expression of *egr3* and total amount of infused oxycodone (Figure 5L).

## Discussion

The current opioid crisis (Boscarino et al., 2010; Rudd et al., 2016; Fornili, 2018) has prompted renewed calls to develop better treatment strategies for patients who misuse these drugs. We have therefore begun to investigate the neurobiological consequences of oxycodone SA behaviors in rats. We have reported recently that rats, given long access to oxycodone, will escalate their intake and show incubation of drug-seeking behaviors after several weeks of withdrawal (Blackwood et al., 2018). As discussed elsewhere (Cadet, 2019), because repeated drug use in humans is not enough to reach a SUD DSM-V diagnosis, we have added contingent footshocks during drug SA to encompass one additional DMS criterion, namely, compulsive drug taking in the presence of adverse consequences. This approach is consistent with the observations that, in clinical situations, only small percentages of individuals continue to misuse drugs when faced with legal and financial consequences (Anthony et al., 1994; Miller and Flaherty, 2000; Santiago Rivera et al., 2018). It needs to be also noted that there exists a substantial number of individuals who use opioids without meeting criteria for OUD (Korf et al., 2010; Zaaijer et al., 2014).

The major findings of the present study include the following: (1) the introduction of contingent punishment to rats that had escalated their intake of oxycodone helped to dichotomize them into two shock-induced phenotypes, with one group of resistant animals that continued to press the active lever for the drug and another group of sensitive rats that stopped or reduced their intake substantially; (2) all oxycodone-exposed rats showed significant decreased striatal protein levels of the three opioid receptors; (3) SS rats showed reduced striatal mRNA expression of several IEGs; whereas (4) *egr3* mRNA expression was significantly increased in the PFC of SR rats. These data are summarized in Tables 2, 3 for readers to refer to. In what follows, we discuss the potential role of some of these changes in gene and protein expression in mediating shock-induced suppression and/or continuation of compulsive oxycodone SA in these rats.

**TABLE 2:** Summary of PCR data from the rat dorsal striatum.

**Gene symbol**	**Gene name**	**SR vs. Sal**	**SS vs. Sal**	**SR vs. SS**
*c-fos*	*Fos proto-oncogene, AP-1 transcription factor subunit*	NS	↓	NS
*fosB*	*FosB proto-oncogene, AP-1 transcription factor subunit*	NS	↓↓	↓
*fra1/fosl1*	*Fos like 1, AP-1 transcription factor subunit*	NS	NS	NS
*fra2/fosl2*	*Fos like 2, AP-1 transcription factor subunit*	↓	↓↓↓	↓
*c-jun*	*Jun proto-oncogene, AP-1 transcription factor subunit*	NS	NS	NS
*junB*	*JunB proto-oncogene, AP-1 transcription factor subunit*	NS	↓	NS
*junD*	*JunD proto-oncogene, AP-1 transcription factor subunit*	NS	NS	NS
*egr1*	*Early growth response 1*	NS	↓↓	NS
*egr2*	*Early growth response 2*	NS	↓↓	↓
*egr3*	*Early growth response 3*	NS	NS	NS

*Arrow points to decreased ↓, IEG expression. Key to arrow: One, Two, Three = *p* < 0.05, 0.01, 0.001, respectively. Non-significant (NS) *p* > 0.05.*

**TABLE 3:** Summary of PCR data from the rat prefrontal cortex.

**Gene symbol**	**Gene name**	**SR vs. Sal**	**SS vs. Sal**	**SR vs. SS**
*c-fos*	*Fos proto-oncogene, AP-1 transcription factor subunit*	NS	NS	NS
*fosB*	*FosB proto-oncogene, AP-1 transcription factor subunit*	NS	NS	NS
*fra1/fosl1*	*Fos like 1, AP-1 transcription factor subunit*	NS	NS	NS
*fra2/fosl2*	*Fos like 2, AP-1 transcription factor subunit*	NS	NS	NS
*c-jun*	*Jun proto-oncogene, AP-1 transcription factor subunit*	NS	NS	NS
*junB*	*JunB proto-oncogene, AP-1 transcription factor subunit*	NS	NS	NS
*junD*	*JunD proto-oncogene, AP-1 transcription factor subunit*	↓	↓↓	NS
*egr1*	*Early growth response 1*	NS	NS	NS
*egr2*	*Early growth response 2*	NS	NS	NS
*egr3*	*Early growth response 3*	↑	NS	↓

*Arrow points to increased ↑, or decreased ↓, IEG expression. Key to arrow: One, Two = *p* < 0.05, 0.01, respectively. Non-significant (NS) *p* > 0.05.*

The present behavioral results are consistent with those of previous papers that had reported that punishment can cause some rats to significantly reduce or stop to self-administer methamphetamine, cocaine, or alcohol (Chen et al., 2013; Marchant et al., 2018; Cadet et al., 2019). The present findings expand the punishment-induced phenomenon to opioids. Our results support the notion that there is not an one-to-one correspondence between escalated drug taking behaviors and a diagnosis of SUD (Cadet, 2019) and supports the idea of using these two phenotypes to attempt to understand the neurobiology of continued drug taking behaviors or abstinence in the presence of adverse consequences.

The present observations of decreased expression of the three opioid receptors in the dorsal striatum suggest that repeated exposure to oxycodone can cause downregulation of these receptors when measured after a short withdrawal interval from oxycodone SA. These findings are consistent, in part, with those of Blackwood et al. (2018) who had reported that the protein levels of only striatal Mu opioid receptors were downregulated in rats that had self-administered oxycodone over a period of 20 days, and that were subsequently withdrawn from the SA experiment for a period of 1 month. When taken together, these observations suggest that there might exist certain regional molecular mechanisms that drive the permanence of the decreased expression of Mu opioid receptors whereas no such mechanisms appear to exist for Delta and Kappa receptors whose expression had returned to normal after 1 month of withdrawal (Blackwood et al., 2018). The relative permanence of decreased levels of striatal Mu receptor protein after long-term exposure to oxycodone might be due, in part, to oxycodone-induced changes in the stability, degradation, or trafficking of these receptors (Williams et al., 2013). These reduced levels might, in part, drive oxycodone SA and/or cue-induced incubation of oxycodone seeking (Blackwood et al., 2018). These mechanisms might not be in play for delta and kappa receptor proteins, of which expression had normalized after a month of oxycodone withdrawal (Blackwood et al., 2018). This idea will need to be tested in future studies using diverse models of OUDs.

It is of interest to note, at this juncture, that clinical studies have reported that several single nucleotide polymorphisms (SNPs) in Mu receptors might influence the effects of oxycodone-induced euphoric responses (Jones et al., 2019) and vulnerability to OUDs (Drakenberg et al., 2006; Nielsen et al., 2008; Levy et al., 2015; Randesi et al., 2016). One SNP of interest to our present discussion is the OPRM A118G, which is associated with increased risk for heroin addiction (Bond et al., 1998; Zhang et al., 2015; see Burns et al., 2019, for a review of other SNPs linked to opioid addiction). This SNP results in reduced maximum binding of the mu opioid receptor ligand, DAMGO, *in vitro* (Bond et al., 1998; Zhang et al., 2005) and in human tissues (Zhang et al., 2005; Weerts et al., 2013). Mice that possess the human equivalent of this SNP have been reported to exhibit decreased expression of both mRNA and protein levels of Mu opioid receptor protein (Mague et al., 2009). Of relevance to the present study, these mice self-administered more heroin than control mice (Zhang et al., 2015). Thus, the possibility exists that the rats that continue to take more oxycodone during footshocks might express a SNP similar to the human OPRM A118G and/or other pro-addiction SNPs. This supposition will need to be tested in future genetic experiments.

Although the changes in the expression of opioid receptors were similar in both resistant and sensitive rats, we found significant differences in the mRNA expression of several IEGs including *fosB*, *fra2*, and *egr2* that showed decreased expression in the dorsal striatum, but not the PFC, of sensitive rats compared to control and resistant rats. It is important to note that the SR and SS phenotypes began to separate almost immediately after the first day of the punishment phase (see Figure 1D). Thus, it is possible to suggest that some of the changes observed in IEG expression might be related to compensatory responses that might have occurred in the SS but not in the SR rats. This argument suggests that there is a need for more studies focusing on quantifying the expression of IEGs at various time points during oxycodone SA and the application of contingent footshocks, once there is measurable evidence of a split between SR and SS rats. It is worth noting that, in our studies of methamphetamine SA and footshocks, we have not been able to identify any effects of footshocks alone on IEG expression (Krasnova et al., 2017; Cadet et al., 2019). It is also possible that rats with differential OPRM1 SNPs might respond differential to contingent footshocks in terms of oxycodone SA and resulting changes in IEG expression during the punishment phase of the study, with the end result being abstention from lever pressing in order to avoid footshocks.

Differential alterations in the expression of several mRNAs and proteins have previously been reported in the dorsal striatum of the SR and SS phenotypes in the case of methamphetamine SA and footshocks (Cadet et al., 2016; Torres et al., 2017). Therefore, we had predicted that the SR rats might show acute increased expression of some IEGs because they were taking oxycodone just 2-h before they were euthanized. We had, indeed, previously found increased expression of *c-fos* in the dorsal striatum of rats that were euthanized after 1 month of withdrawal from escalated oxycodone intake (Blackwood et al., 2019). We also thought that the levels of expression of IEGs might be normal or might be reduced in the SS group in comparison to the SR rats. We found, however, that striatal IEG mRNA levels were mostly decreased in the SS animals without there being any major changes in the SR rats. Of all our observations, the decreases in *fosB* mRNA expression in the dorsal striatum are of interest because *fosB* expression has been reported to be altered after exposure to methamphetamine (Krasnova et al., 2013; Cadet et al., 2015), cocaine (Hope et al., 1992; Larson et al., 2010), opioid receptor agonists (Liu et al., 1994), and nicotine (Saint-Preux et al., 2013), with most papers reporting increased *fosB* expression. Thus, our findings of decreased *fosB* in the shock-sensitive rats suggest that reduced *fosB* expression might participate in a molecular cascade that helps to suppress oxycodone-taking behaviors, possibly via a SNP-dependent fashion (see section “Discussion” above). This conclusion is consistent with the report that overexpression of delta *fosB*, an alternative splicing product of *fosB* mRNA in which Exon IV is truncated (Alibhai et al., 2007), can enhance the rewarding effects of cocaine when mice were tested in the conditioned place preference procedure (Kelz et al., 1999). Because these authors used cocaine in that study, the effects of this genetic manipulation will need to be tested in future oxycodone SA experiments. It is important to indicate that there are several fosB target genes that may be relevant to this discussion (Nestler, 2015). These include the glutamate AMPA receptor, GluA2/GluR2, the opioid peptide, dynorphin, and CAMKIIalpha that have been implicated in models of SUDs (Nestler, 2015).

Although the above discussion has focused on the potential role of decreased FosB and/or delta FosB in suppressing oxycodone SA, similar arguments could be put forward for *fra2* and *egr2* that also showed significant decreases in the dorsal striatum of sensitive rats in comparison to the resistant animals. It is also of interest that the changes in *fosB*, *fra2*, and *egr2* mRNA levels occurred only in the dorsal striatum, further implicating that brain region in the manifestation of compulsive habit-like behaviors (Everitt and Robbins, 2016; Hodebourg et al., 2018) in oxycodone self-administering rats (Blackwood et al., 2018). Although this discussion has focused on IEG expression, several recent papers on oxycodone self-administration have identified other genes that might be relevant to oxycodone use disorder (Zhang et al., 2014, 2017, 2018). These included mRNAs coding for GABA-, glutamate, and dopamine receptors that were downregulated in the dorsal striatum (Zhang et al., 2018). In contrast, NPY5 receptor and the glycine receptor, alpha 4 subunit, were reported to show increases after oxycodone SA in the dorsal striatum of mice (Zhang et al., 2015). In addition, some structural genes, including integrins were also affected in the dorsal striatum of oxycodone self-administering mice (Yuferov et al., 2018). Because these authors had used all oxycodone-exposed animals in their studies, it will be important to test whether the shock-induced SR and SS phenotypes will show differential expression of some of these genes.

It is also noteworthy that *egr3* (Patwardhan et al., 1991) was the only gene that showed increased mRNA expression in the PFC of resistant rats in comparison to control and sensitive rats. Egr3 expression is rapidly regulated in hippocampal and cortical neurons by electroconvulsive seizures (Yamagata et al., 1994). Changes in the expression of this IEG may be relevant to the effects of drugs of abuse because *egr3* mRNA expression is induced by acute administration of psychostimulants including cocaine and methamphetamine (O’Donovan and Baraban, 1999; Courtin et al., 2006; McCoy et al., 2011). Repeated injections of cocaine also induced Egr3 expression (Jouvert et al., 2002) in D1-containing neurons in the ventral striatum (Chandra et al., 2015). Importantly, Egr3 knockdown in D1-containing neurons reduced cocaine-associated conditioned place preference (CPP) and cocaine-induced locomotor hyperactivity. Taken together, our observations of increased *egr3* expression in the PFC of rats that took oxycodone compulsively extends the role of Egr3 to opioid-driven behaviors, possibly, by regulating executive functions (Duverne and Koechlin, 2017; Jayachandran et al., 2019) that may include decisions to continue to take oxycodone compulsively in the presence of adverse consequences.

## Conclusion

The present study provides the first evidence that contingent footshocks can dichotomize oxycodone SA rats into two phenotypes of resistant and sensitive animals in a fashion similar to what has been reported for alcohol, cocaine, and methamphetamine. Our findings of significant decreases in striatal *fosB*, *fra2*, and *egr2* mRNA levels in the sensitive in comparison to the resistant rats suggest that IEGs of diverse classes might participate in molecular networks that drive long-term changes in striatal neuronal structures and functions that might serve to suppress habitual drug-taking behaviors. In contrast, increased *egr3* expression in the PFC, a structure that has been implicated in decision making and memory functions (Duverne and Koechlin, 2017; Jayachandran et al., 2019), supports the notion that Egr3 may regulate rewarding effects of psychostimulants (Chandra et al., 2015) and the propensity to continue to take oxycodone in the presence of punishment. These ideas will need to be tested to identify the specific roles that these diverse IEGs might play in the regulation of punishment-induced abstinence or compulsive oxycodone taking.

## Data Availability Statement

All datasets generated for this study are included in the article/supplementary material.

## Ethics Statement

The animal study was reviewed and approved by the NIDA (National Institute of Drug Abuse) Animal Care and Use Committee at the Intramural Research Program (IRP).

## Author Conributions

CB, MM, and BL performed self-administration and punishment test. CB and MM performed western blot and RT-qPCR experiments. CB and JC prepared the manuscript. JC supervised the overall project.

## Conflict of Interest

The authors declare that the research was conducted in the absence of any commercial or financial relationships that could be construed as a potential conflict of interest.

## Footnotes

1 https://grants.nih.gov/grants/olaw/guide-for-the-care-and-use-of-laboratory-animals.pdf
